# Stopping MYC in its tracks

**DOI:** 10.18632/aging.100780

**Published:** 2015-07-16

**Authors:** Eduard Stefan, Jonathan R. Hart, Klaus Bister

**Affiliations:** Institute of Biochemistry and Center for Molecular Biosciences (CMBI), University of Innsbruck, Austria

**Figure 1:**
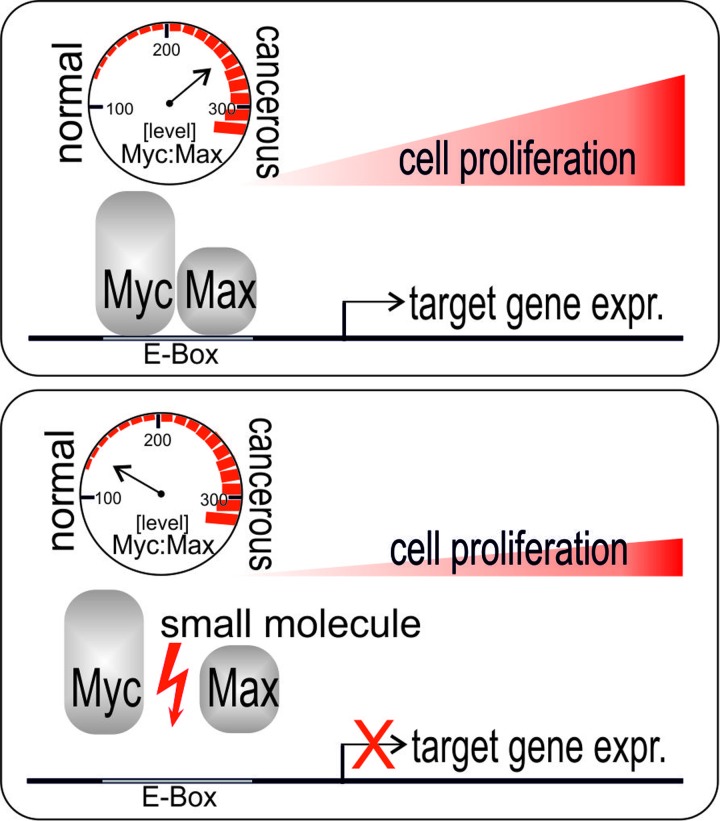
Elevated levels of MYC-MAX complexes drive cell proliferation and carcinogenesis The oncoprotein MYC and its dimerization partner MAX bind to specific DNA motifs (E-Box) and control the expression of a vast array of target genes. Elevated MYC levels reprogram target gene expression profiles which promote the cancer state. Small-molecule inhibitors of MYC-MAX protein-protein interaction reduce transcription factor binding to DNA and thus interfere with MYC-driven cancer cell proliferation.

MYC is a transcriptional regulator that in order to function has to dimerize with a specific partner protein, MAX. The MYC-MAX dimer regulates the expression of thousands of genes involved in fundamental cellular processes including growth, proliferation, differentiation, biosynthesis, energy metabolism, and apoptosis [1-3]. The discovery that MYC becomes overexpressed as a result of chromosomal re-arrangements in Burkitt's lymphoma was the first implication of MYC in human cancer, and today deregulated MYC expression is considered to be the crucial driving force in most if not all cancers [1-3]. Because of this prevalent role in carcinogenesis, MYC is an important target for therapeutic intervention. However, there are both principal and practical obstacles in targeting MYC. Inhibition of a gene that is essential for fundamental cellular processes could cause unacceptable side effects. Yet inhibition of MYC by expression of a dominant-negative MYC construct in an animal model caused regression of tumor growth but no lasting damage to rapidly proliferating normal tissues [4]. Practical problems in directly targeting MYC or the MYC-MAX heterodimer with small molecules (Figure 1) stem from the disordered state of the MYC monomer in solution and from the general nature of protein-protein interactions. These commonly involve large interacting surfaces that present no well-defined pockets or grooves for high-energy binding of small ligands. However, proof of principle for overcoming these difficulties was provided by the identification of small-molecule antagonists for MYC-MAX dimerization that reduced MYC-driven cell transformation in tissue culture [5].

Two recent publications in *PNAS* and *Oncotarget* now report the identification and characterization of novel small-molecule inhibitors of MYC-MAX dimerization (Figure 1) that are active in a pharmacologically relevant nanomolar range [6,7]. The MYC-MAX antagonists were isolated from a Kröhnke combinatorial library of 2,4,6-trisubstituted pyridines designed for drug discovery. These lead compounds inhibit MYC-MAX dimerization, specifically interfere with MYC-induced oncogenic transformation in cell culture, reduce the MYC-specific transcriptional signature, and block MYC-driven tumor growth in a xenotransplant of human cancer cells [6]. These data were complemented with a specific protein-fragment complementation assay (PCA). In this assay, *Renilla* luciferase (*R*luc) is rationally dissected into two fragments, one of these is fused to MYC, the other to MAX. When the MYC and MAX components of these hybrid proteins dimerize, luciferase activity is restored. This PCA allows direct recording of the interplay of MYC and MAX in living cells [7]. The studies documented inhibition of MYC-MAX dimerization by the small-molecule inhibitors, showed the expected nuclear localization of MYC-MAX complexes, and demonstrated the effect of inactivating MYC mutations on the nuclear MYC-MAX complex levels as well as sensitivity of MYC-MAX dimerization to limiting levels of available MAX. The degree to which MYC-MAX levels are reduced by the small-molecule antagonists correlates with the cytocidal and cytostatic activity of the inhibitors for MYC-driven human or avian tumor cells. The *R*luc PCA is a specific and sensitive reporter assay broadly applicable to the analysis of protein-protein interactions, including screening and optimization of small-molecule inhibitors. The promising features of the MYC inhibitors described in the two recent reports [6,7] will initiate further efforts to improve their pharmacokinetic properties, and to unveil their precise binding mode and molecular mechanism of interference with MYC-MAX function.
